# Empirical glycopeptide exposure and acute kidney injury during cloxacillin therapy in *Staphylococcus aureus* bacteremia

**DOI:** 10.1128/aac.00128-26

**Published:** 2026-05-04

**Authors:** Miguel Ángel Verdejo, Daniela Malano-Barletta, Cristina Pitart, Marta Hernández-Meneses, Guillermo Cuervo, Marta Bodro, Carolina García-Vidal, Néstor Lopez, Sabina Herrera, Ana del Río, Mateu Espasa, Climent Casals, Josep Mensa, Laura Morata, Alex Soriano

**Affiliations:** 1 Infectious Diseases Department, Hospital Clínic Barcelona 16493, Barcelona, Spain; 2 Institut d’Investigacions Biomèdiques August Pi i Sunyer (IDIBAPS) 146245 , Barcelona, Spain; 3 Faculty of Medicine, University of Barcelona 16724, Barcelona, Spain; 4 Microbiology Department, Hospital Clínic Barcelona–University of Barcelona 16724, Barcelona, Spain; 5 CIBERINFEC, CIBER in Infectious Diseases, ISCIII, Madrid, Spain; The Peter Doherty Institute for Infection and Immunity, Melbourne, Victoria, Australia

**Keywords:** *Staphylococcus aureus *bacteremia, acute kidney injury, nephrotoxicity, cloxacillin, cefazolin, glycopeptides

## Abstract

We aimed to identify risk factors for the development of acute kidney injury (AKI) during treatment of *Staphylococcus aureus* bacteremia (SAB), with particular emphasis on the roles of cloxacillin and cefazolin, and the contribution of other antibiotics. We performed a retrospective, observational, single-center study of a prospective cohort of patients with SAB receiving cloxacillin or cefazolin as definitive therapy and with serum creatinine available at baseline and days 5, 10, and 15. The primary outcome was the highest AKI stage between days 5 and 15; the secondary outcome was 30 day mortality. Univariate and multivariate analyses were performed to identify independent predictors of outcomes. A total of 470 patients were included in the study, and 103 (21.9%) developed AKI during hospitalization. Independent predictors of AKI were age >64 years (adjusted odds ratio [aOR] 1.76; 95% CI 1.08–2.87), peripheral arterial disease (aOR 2.23; 95% CI 1.02–4.88), chronic kidney disease (aOR 3.77; 95% CI 2.07–6.89), need of mechanical ventilation (aOR 3.33; 95% CI 1.62–6.86), and cloxacillin as definitive treatment with prior glycopeptide exposure (aOR 3.45; 95% CI 1.74–6.84). AKI occurred in 42.6% (23/52) of patients receiving empirical glycopeptide followed by cloxacillin, compared with 21.0% (61/290) in those treated with cloxacillin without prior glycopeptide exposure. The development of AKI during admission was an independent predictor of 30 day mortality (aOR 3.50; 95% CI 1.88–6.52). The only modifiable factor associated with AKI was the use of cloxacillin as definitive therapy after prior exposure to glycopeptides. These data reinforce the need to consider non-nephrotoxic alternatives.

## INTRODUCTION

Development of nephrotoxicity, even when subclinical, has a severe impact on clinical outcomes in critically ill patients (1). Acute kidney injury (AKI) is a frequent complication in *Staphylococcus aureus* bacteremia (SAB) and is associated with higher mortality (2). The backbone of therapy for methicillin-susceptible *S. aureus* (MSSA) continues to rely on semisynthetic penicillins (nafcillin, cloxacillin, flucloxacillin) or cefazolin (3). However, recent data showing a higher risk of nephrotoxicity associated with semisynthetic penicillins, either as monotherapy or in combination with vancomycin, may tip the balance toward the use of cefazolin (4–6).

At our institution, cloxacillin has traditionally been the backbone of therapy for MSSA bacteremia, based on evidence from animal models and clinical studies suggesting that its activity is not affected by the inoculum effect (7–9). However, emerging data on the potential nephrotoxicity of cloxacillin prompted a revision of our institutional protocol. Since 2021, cefazolin has therefore been adopted as the preferred β-lactam for the treatment of MSSA bacteremia.

In the present study, our objective was to identify risk factors for the development of AKI during the first 15 days following *S. aureus* bacteremia, as assessed by changes in serum creatinine, with particular emphasis on the potential roles of cloxacillin and cefazolin as definitive therapy, as well as the contribution of antibiotics used as part of empirical treatment.

## MATERIALS AND METHODS

### Study design

We conducted a retrospective, observational, single-center study of our prospectively identified *S. aureus* bacteremia cohort at Hospital Clínic Barcelona, Spain, a tertiary care university hospital with approximately 800 inpatient beds. All adult patients (≥16 years old) admitted to our hospital with at least one positive blood culture for *S. aureus* and signs or symptoms of infection, between January 2006 and December 2024, were included. For the present analysis, only patients with MSSA bacteremia who received cloxacillin or cefazolin as definitive therapy and with data on serum creatinine at baseline (day of blood culture), and at day 5, 10, and 15 (±1 day), were included. These creatinine cut-off points were chosen in order to track changes in creatinine levels over the first 2 weeks of treatment for SAB. This study focused on AKI developing from *S. aureus* bacteremia onward, as assessed by changes in serum creatinine, because these events are potentially related to antimicrobial exposure. All patients had at least the first creatinine follow-up (day 5); therefore, those dying, discharged, or transferred to another hospital before day 5 were excluded from the analysis. Patients under chronic renal replacement therapy (hemodialysis or peritoneal dialysis) were also excluded.

### Data collection

Our cohort has been described previously (10). Briefly, every positive blood culture for *S. aureus* is systematically reported to the Infectious Diseases (ID) team, who evaluates each case and prospectively records relevant demographic, laboratory, microbiological, and clinical data in a hospital-wide registry using a standardized report form. Serum creatinine concentrations were later extracted from Electronic Medical Records (EMR) by ID specialists from the Nosocomial Infection Research Group. Collected variables included age, gender, whether the infection was community-acquired vs healthcare-related, and comorbidities such as diabetes, cardiac diseases including prosthetic valve or intracardiac devices, chronic respiratory disease, chronic kidney disease (documented pre-existing renal impairment), dialysis dependence, liver cirrhosis, alcohol consumption, solid organ or hematological neoplasia under active treatment, transplant recipients, or other causes of immunosuppression. For the purposes of this study, individual comorbidities were evaluated rather than the composite Charlson score, given that the risk of AKI differs substantially across specific conditions (e.g., chronic kidney disease or chronic respiratory disease). Clinical data encompassed shock at presentation, admission to intensive care unit (ICU) or need for mechanical ventilation, infection foci, presence of septic metastases, appropriate empiric antibiotic therapy, and details about empiric and definitive antibiotic therapy. Microbiologic data included first and consecutive blood cultures.

The origin of bacteremia was categorized into community-acquired according to previous definitions (11). Persistent bacteremia was considered when blood cultures remained positive after ≥48 h of active anti-staphylococcal therapy (12). The foci of infection were categorized by mortality risk (≥15% or <15%). Empirical (first 24 h from blood culture obtention) and definitive (according to identification and antibiogram) antibiotics with activity against MSSA were evaluated and grouped as aminoglycosides (gentamicin, tobramycin, or amikacin), glycopeptides (vancomycin or teicoplanin), daptomycin, carbapenems (imipenem, meropenem, or ertapenem), piperacillin-tazobactam, third-generation cephalosporins (ceftriaxone or cefotaxime), cloxacillin, and cefazolin.

### Outcome measurement

The primary outcome was the highest AKI stage between days 5 and 15 after *S. aureus* bacteremia. AKI was calculated in reference to the baseline value for the three time points (at day 5, 10, and 15) and was categorized into three stages as follows: I, when there was an increase of ≥0.3 mg/dL or a ratio ≥1.5 but <2; II, when the ratio was ≥2 but <3; and III, when the ratio was ≥3 or creatinine ≥4 mg/dL. As urinary output was not consistently recorded in EMR, it was not considered when identifying AKI retrospectively. The secondary outcome was the 30 day all-cause mortality.

### Statistical analysis

Categorical data were described as absolute count and percentage, and quantitative data as mean and standard deviation or median and interquartile range (IQR) (Q1–Q3). Comparisons of categorical variables were done with the χ^2^ test or Fisher’s test when appropriate.

We calculated AKI as previously described at each time point (days 5, 10, or 15). Then, a binary dependent variable was created, classifying patients as having developed AKI (stages I–III) or not at any time point. A multivariable logistic regression model was developed to assess the association of definitive therapy with cloxacillin or cefazolin and the development of AKI. The model was adjusted for potential confounders in bivariate analysis (*P* < 0.05), including cardiovascular disease, diabetes mellitus, chronic renal failure, respiratory disease, liver cirrhosis, malignancy, immunosuppression, septic shock, need for ICU or mechanical ventilation, inappropriate empirical treatment, baseline creatinine, and high-risk infection foci. Age was dichotomized by the median value to facilitate model interpretation. The same modeling approach was used for the secondary outcome of 30 day mortality. The model included variables associated with mortality in the bivariate analysis, including the development of AKI during SAB treatment.

Interaction terms were assessed by inclusion in the multivariable model, guided by clinical plausibility and exploratory stratified analyses; non-significant interactions were excluded for the final model. Independent variables were selected for the final model using a backward stepwise elimination procedure based on the Wald test. The results are presented as adjusted odds ratios (aORs) with 95% confidence intervals (CIs). The goodness-of-fit of the final model was evaluated using the Hosmer-Lemeshow test. All statistical analyses were performed using SPSS version 30.0.

## RESULTS

A total of 470 patients were included in the study, with a mean (SD) age of 63.1 (16.1) years; 34.9% were female. The median (IQR) Charlson comorbidity index was 2 (1–4). Infection was classified as community-acquired in 206 cases (43.8%). The most frequent source of bacteremia was an intravascular catheter, identified in 181 patients (38.5%). Septic shock at admission was present in 66 patients (14.0%). The median (IQR) baseline serum creatinine was 1.1 mg/dL (0.8–1.7). Cloxacillin was administered as definitive antimicrobial therapy to 344 patients (73.2%), while cefazolin was given to 126 patients (26.8%). Overall, 103 patients (21.9%) met criteria for AKI during hospitalization: 65 (13.8%) developed AKI stage I, and 38 (8.1%) developed AKI stage II or III. The 30 day all-cause mortality rate was 11.7%.

Table 1 summarizes patient characteristics according to the development of acute kidney injury during hospitalization. Patient-related factors associated with AKI included older age and the presence of diabetes mellitus, chronic heart failure, ischemic heart disease, peripheral arterial disease, cerebrovascular disease, and chronic kidney disease. Indicators of greater infection severity associated with AKI were higher baseline serum creatinine at the time of blood culture collection, need for ICU admission, and requirement for mechanical ventilation. With respect to antimicrobial exposure, the use of glycopeptides as empirical therapy and the choice of cloxacillin versus cefazolin as definitive therapy were both significantly associated with the development of AKI.

**TABLE 1:** Characteristics of patients with MSSA bacteremia according to the development of AKI during admission^*a*^

Variable	No AKI (*n* = 367)	AKI (*n* = 103)	*P*-value
Patient-related factors			
Age ≥64 years	170 (46.4)	64 (62.7)	0.005
Female	132 (36)	32 (31)	0.413
Community-acquired infection	163 (44.5)	43 (41.7)	0.650
Diabetes mellitus	93 (25.3)	46 (44.7)	<0.001
Ischemic heart disease	55 (15)	25 (24.3)	0.037
Cardiac valvular disease	53 (14.4)	19 (18.4)	0.353
Prosthetic valve	26 (6.8)	9 (8.7)	0.520
Chronic heart failure	31 (8.5)	23 (22.3)	<0.001
CIED	18 (4.9)	9 (8.7)	0.153
Peripheral artery disease	21 (5.8)	14 (13.6)	0.003
Cerebrovascular disease	25 (6.8)	14 (13.6)	0.041
Respiratory disease	49 (13.4)	14 (13.6)	1
Chronic kidney disease	39 (10.7)	31 (30.1)	<0.001
Liver cirrhosis	38 (10.4)	9 (8.7)	0.713
Solid neoplasm	87 (23.8)	19 (18.4)	0.287
Hematological neoplasm	25 (6.8)	2 (1.9)	0.090
Solid organ transplant	14 (3.8)	4 (3.9)	1
Hematopoietic stem cell transplant	2 (0.5)	0	1
Neutropenia (≤500 cells/mL)	10 (2.7)	1 (1)	0.469
Steroid therapy	48 (13.1)	15 (14.6)	0.744
HIV infection	19 (5.2)	2 (1.9)	0.277
Indicators of infection severity			
Foci^*b*^			
Intravenous catheter	135 (36.8)	46 (44.7)	0.169
Osteo-articular infection	53 (14.4)	8 (7.8)	0.096
SSTI	51 (13.9)	9 (8.7)	0.185
Unknown	45 (12.3)	14 (13.6)	0.737
Endocarditis	28 (7.6)	11 (10.7)	0.316
Pneumonia	9 (2.5)	6 (5.8)	0.109
Urinary tract infection	11 (3)	2 (1.9)	0.743
Septic metastasis	108 (29.4)	32 (31.1)	0.807
Septic shock	48 (13.1)	18 (17.5)	0.263
Need of ICU	72 (19.7)	33 (32.4)	0.010
Need of MV	25 (6.8)	17 (16.7)	0.005
Baseline creatinine ≥1.10 mg/dL	156 (42.5)	72 (69.9)	<0.001
Persistent bacteremia	99 (27)	37 (35.9)	0.086
Antimicrobial therapy			
Active empiric therapy	306 (85.2)	81 (80.2)	0.221
Empiric therapy^*d*^			
Aminoglycoside	9 (2.5)	4 (3.9)	0.495
Glycopeptide^*c*^	37 (10.1)	23 (22.3)	0.002
Daptomycin	119 (32.4)	30 (29.1)	0.551
PTZ	54 (14.7)	12 (11.6)	0.520
Carbapenem	77 (21)	25 (24.3)	0.500
3GC	87 (23.7)	18 (17.5)	0.228
Cloxacillin	47 (12.8)	10 (9.7)	0.495
Definitive therapy			
Cloxacillin	260 (70.8)	84 (81.6)	0.032
Cefazolin	107 (29.2)	19 (18.4)	0.032

^*a*^ AKI, acute kidney injury. CIED, cardiac implantable electronic device. SSTI, skin and soft tissue infection. ICU, intensive care unit. MV, mechanical ventilation. PTZ, piperacillin-tazobactam. 3GC, third generation cephalosporin.

^*b*^ Those with >10 cases are included in the list.

^*c*^ This group included 38 with vancomycin (63%) and 22 with teicoplanin (37%).

^*d*^ Total exceeds 100% because a patient could receive more than one empirical antibiotic.

In multivariable analysis, independent predictors of AKI were age >64 years (aOR 1.76; 95% CI 1.08–2.87), peripheral arterial disease (aOR 2.23; 95% CI 1.02–4.88), chronic kidney disease (aOR 3.77; 95% CI 2.07–6.89), the need for mechanical ventilation (aOR 3.33; 95% CI 1.62–6.86), and cloxacillin as definitive treatment with prior glycopeptide exposure (aOR 3.45; 95% CI 1.74–6.84) (Table 2).

**TABLE 2:** Variables independently associated with developing AKI during the admission

Variable	Adjusted OR (95% CI)	*P*-value
Age ≥64 years	1.75 (1.07–2.87)	0.025
Peripheral arterial disease	2.22 (1.01–4.87)	0.045
Chronic kidney disease	3.77 (2.06–6.88)	<0.001
Need of mechanical ventilation	3.33 (1.61–6.85)	0.001
Antibiotic exposure:		
Cefazolin as definitive treatment	Ref.	1
Cloxacillin as definitive treatment without prior glycopeptide	0.75 (0.41–1.36)	0.350
Cloxacillin as definitive treatment with prior glycopeptide	3.45 (1.74–6.83)	<0.001

The incidence of AKI among patients who received empirical glycopeptide therapy followed by cloxacillin as definitive treatment was 42.6% (23/52), compared with 21.0% (61/290) among those patients treated with cloxacillin who did not receive empirical glycopeptides (*P* = 0.002). Because vancomycin was progressively discontinued at our institution, only a small number of patients receiving cefazolin as definitive therapy had been exposed to empirical glycopeptides (*n* = 6), and none developed AKI. Among patients who did not receive empirical glycopeptides and were treated definitively with cefazolin, the AKI rate was 15.8% (19/120), which was slightly inferior but not significantly different to that observed in patients receiving cloxacillin without empirical glycopeptides (21.0%; *P* = 0.270) (Table 3). The temporal evolution of AKI stages at days 5, 10, and 15, stratified by treatment group, is presented in Supplementary material (Table S1).

**TABLE 3:** Ratio of AKI during admission according to definitive anti-staphylococcal therapy and the prior exposure to glycopeptides (in parenthesis, the percentage of the rows)^*a*^

Variable	No AKI (%)	AKI (I–III) (%)	AKI I (%)	AKI II–III (%)
No empiric glycopeptide:				
Definitive cloxacillin	229 (79.0)	61 (21.0)^a^	42 (14.7)	19 (6.5)
Definitive cefazolin	101 (84.2)	19 (15.8)^b^	11 (9.1)	8 (6.6)
Empiric glycopeptide:				
Definitive cloxacillin	31 (57.4)	23 (42.6)^c^	12 (20.7)	11 (20.3)
Definitive cefazolin	6 (100.0)	0 (0.0)	0	0

^*a*^ Chi-squared test, a vs b, *P *= 0.270; a vs c, *P *= 0.002.

Thirty-day mortality was evaluated as a secondary outcome. In univariable analysis, factors associated with mortality included age ≥64 years, infection source, septic shock, need for ICU admission, requirement for mechanical ventilation, baseline serum creatinine ≥1.1 mg/dL, and development of AKI during hospitalization. In multivariable analysis, independent predictors of 30 day mortality were age ≥64 years (aOR 2.05; 95% CI 1.08–3.90), septic shock (aOR 4.24; 95% CI 2.16–8.33), high-risk foci of infection (aOR 2.19; 95% CI 1.18–4.05), and development of AKI during admission (aOR 3.50; 95% CI 1.88–6.52) (Table S2).

## DISCUSSION

In this study, 21.9% of patients with MSSA bacteremia developed acute kidney injury during hospitalization, and the only modifiable factor associated with AKI was the use of cloxacillin as definitive therapy in patients with prior exposure to glycopeptides as empirical treatment. While others reported a higher rate of AKI because they considered both early renal failure, using a prior creatinine value for comparison with the baseline value, and late renal failure, comparing the baseline value with subsequent values (2, 13), we focused on AKI that developed during treatment for *S. aureus* bacteremia, with the aim of evaluating the impact of antibiotics on renal function. This is clinically relevant given the well-recognized impact of renal dysfunction on patient outcomes, a fact that was also confirmed in our cohort. After adjustment for major predictors of mortality (age, infection severity, and infection source) and baseline creatinine, the worsening of renal function during admission was independently associated with a 3.5-fold increase in 30 day mortality.

These observations highlight the importance of identifying potentially modifiable risk factors contributing to renal injury. In this context, isoxazolic penicillins have recently been implicated as possible contributors to nephrotoxicity. However, the evidence remains heterogeneous. In a recent systematic review (4), the reported increased risk of nephrotoxicity associated with anti-staphylococcal penicillins was largely driven by studies evaluating nafcillin, a drug with well-recognized nephrotoxic potential. Only two studies included other anti-staphylococcal penicillins. Lecomte et al. (14) evaluated 157 cases of infective endocarditis treated with cloxacillin and 53 treated with cefazolin, reporting renal failure in seven patients (4.4%) in the cloxacillin group and none in the cefazolin group; however, renal failure was not formally defined. In contrast, Lefevre et al. (15), using a standardized definition of AKI, did not observe significant differences between patients treated with anti-staphylococcal penicillins (*n* = 35) and those receiving cefazolin (*n* = 38). More recently, a prospective multicenter study from the Netherlands including 453 episodes of *S. aureus* bacteremia identified risk factors for AKI similar to those observed in our cohort and found vancomycin to be the only antimicrobial independently associated with AKI, whereas flucloxacillin and aminoglycosides were not. However, the potential interaction between empirical vancomycin exposure and subsequent flucloxacillin therapy was not assessed (16).

In our cohort, glycopeptides were administered as empirical therapy, in general for 24–48 h before switching to definitive therapy with cloxacillin or cefazolin. The main finding of our study is that patients who received cloxacillin as definitive therapy exhibited a higher risk of AKI than those treated with cefazolin; however, this increased risk was confined to patients who had previously been exposed to glycopeptides as part of empirical therapy. The incidence of AKI was 15.8% among patients treated with cefazolin, 21.0% among those receiving cloxacillin without prior glycopeptide exposure, and 42.6% among those receiving cloxacillin following empirical glycopeptide therapy. In multivariate analysis, empiric glycopeptide therapy followed by definitive cloxacillin remained significantly associated with AKI (aOR 3.45; 95% CI 1.74–6.84). Therefore, antibiotic-associated nephrotoxicity in *S. aureus* bacteremia appears to depend not only on the definitive agent selected but also on the sequence of antimicrobial exposure. Vancomycin, a highly hydrophilic molecule (logP ≈ −3), accumulates in proximal tubular epithelial cells through receptor-mediated endocytosis from the urine and transporter-mediated secretion from peritubular circulation, inducing mitochondrial dysfunction and oxidative stress even after short exposures (17). This initial tubular injury may be subclinical and not immediately reflected by changes in serum creatinine. In contrast, isoxazolic penicillins are clearly lipophilic molecules (logP ~ +2–3), with a propensity for intracellular accumulation and the potential to exacerbate pre-existing tubular vulnerability (18). Within this framework, our findings support a model of sequential nephrotoxicity, whereby prior, even brief, exposure to glycopeptides predisposes patients to the development of AKI when definitive therapy with cloxacillin is subsequently initiated. This effect was not observed with cefazolin, a more hydrophilic cephalosporin, suggesting that the lipophilicity of the definitive antibiotic may modulate renal risk following an initial tubular insult.

Two recent clinical trials compared cloxacillin and cefazolin for the treatment of *S. aureus* bacteremia (6, 19), and in both studies, the incidence of AKI was significantly higher in the cloxacillin arm. However, it is important to note that randomization was permitted within the first 3 days after diagnosis, during which time a proportion of patients were likely receiving glycopeptides as empirical therapy. From this perspective, it remains essential to clarify whether the increased risk of AKI attributed to cloxacillin is confined to patients with prior glycopeptide exposure.

The main limitation of this study is its retrospective design, which carries an inherent risk of unmeasured confounding. Nevertheless, the cohort was assembled in a single center by infectious diseases specialists using a standardized approach, which supports the consistency and reliability of the collected data. A second important limitation is the lack of systematic data on exposure to other potentially nephrotoxic agents, such as diuretics or nonsteroidal anti-inflammatory drugs, which are frequently used in this population and could have influenced the observed risk of AKI. Third, AKI was assessed using changes in serum creatinine, a parameter with recognized limitations; however, its strong and independent association with mortality in our cohort supports its clinical relevance. Although KDIGO creatinine thresholds were applied, the timing of measurements did not strictly adhere to KDIGO criteria. The secondary outcome of 30 day mortality should be interpreted bearing in mind that patients who died or were discharged before the first follow-up creatinine value (5 days) were excluded from the analysis. Finally, confirmation of an additive effect between short-term glycopeptide exposure and cloxacillin on renal injury would require a larger comparator group. Only six patients received cefazolin following prior glycopeptide exposure, none of whom developed AKI. Although this finding is noteworthy, larger studies are needed to validate this observation.

In conclusion, 21.9% of patients with *S. aureus* bacteremia developed AKI during hospitalization, and AKI was independently associated with increased 30 day mortality. The only potentially modifiable factor associated with AKI was the use of cloxacillin as definitive therapy in patients with prior exposure to glycopeptides for 24–48 h. From a clinical perspective, these data reinforce the need to minimize empirical exposure to glycopeptides and to consider non-nephrotoxic alternatives when available, particularly in patients with underlying renal risk factors.

## Data Availability

The data supporting the findings of this study are available from the corresponding author upon reasonable request.

## SUPPLEMENTAL MATERIAL

Supplemental material aac.00128-26-s0001.docx Tables S1 and S2.

ASM does not own the copyrights to Supplemental Material that may be linked to, or accessed through, an article. The authors have granted ASM a non-exclusive, world-wide license to publish the Supplemental Material files. Please contact the corresponding author directly for reuse.
