# Supplementary material for: Empirical glycopeptide exposure and acute kidney injury during cloxacillin therapy in Staphylococcus aureus bacteremia
Source: Antimicrob Agents Chemother. 2026 May 4;70(6):e00128-26. doi: 10.1128/aac.00128-26 (PMC13231869; doi:10.1128/aac.00128-26)
Supplement: Supplemental material — Tables S1 and S2. [file aac.00128-26-s0001.docx]

**SUPPLEMENTAL MATERIAL**

Table S1. Evolution of AKI stages at days 5, 10, and 15 according to definitive anti-staphylococcal therapy and prior exposure to glycopeptides.

|  |  | No AKI  n/N (%) | AKI I  n/N (%) | AKI II  n/N (%) | AKI III  n/N (%) |
| --- | --- | --- | --- | --- | --- |
| Cefazolin as definitive therapy without prior glucopeptyde exposure | Day 5 | 92/106 (86.8) | 7/106 (6.6) | 0/106 (0.0) | 7/106 (6.6) |
|  | Day 10 | 84/91 (92.3) | 4/91 (4.4) | 0/91 (0.0) | 3/91 (3.3) |
|  | Day 15 | 63/67 (94.0) | 3/67 (4.5) | 0/67 (0.0) | 1/67 (1.5) |
| Cefazolin as definitive therapy with prior glucopeptyde exposure | Day 5 | 5/5 (100.0) | 0/5 (0.0) | 0/5 (0.0) | 0/5 (0.0) |
|  | Day 10 | 5/5 (100.0) | 0/5 (0.0) | 0/5 (0.0) | 0/5 (0.0) |
|  | Day 15 | 4/4 (100.0) | 0/4 (0.0) | 0/4 (0.0) | 0/4 (0.0) |
| Cloxacillin as definitive therapy without prior glucopeptyde exposure | Day 5 | 233/275 (84.7) | 28/275 (10.2) | 4/275 (1.5) | 10/275 (3.6) |
|  | Day 10 | 216/243 (88.9) | 18/243 (7.4) | 5/243 (2.1) | 4/243 (1.6) |
|  | Day 15 | 176/205 (85.9) | 19/205 (9.3) | 4/205 (2.0) | 6/205 (2.9) |
| Cloxacillin as definitive therapy with prior glucopeptyde exposure | Day 5 | 38/53 (71.7) | 7/53 (13.2) | 1/53 (1.9) | 7/53 (13.2) |
|  | Day 10 | 37/51 (72.5) | 6/51 (11.8) | 3/51 (5.9) | 5/51 (9.8) |
|  | Day 15 | 38/46 (82.6) | 6/46 (13.0) | 0/46 (0.0) | 2/46 (4.3) |

AKI: acute kidney injury.

Table S2. Patient characteristics and multivariate predictors of 30-day mortality in MSSA bacteraemia.

| **Variable** | **Alive (N=413)** | **Dead* (N=55)** | **p-value** | **Multivariate analysis**  **aOR (CI95%)** | **p-value** |
| --- | --- | --- | --- | --- | --- |
| **Age ≥64 years** | **195 (47.4)** | **37 (67.3)** | **0.006** | **2.04 (1.07-3.87)** | **0.029** |
| Female | 138 (33.4) | 25 (45.5) | 0.097 |  |  |
| Community-acquired | 177 (43) | 28 (50.9) |  |  |  |
| Diabetes mellitus | 119 (28.8) | 18 (32.7) | 0.532 |  |  |
| Ischemic heart disease | 68 (16.5) | 12 (21.8) | 0.341 |  |  |
| Heart valve disease | 64 (15.5) | 8 (14.5) | 1 |  |  |
| Prosthetic valve | 30 (7.3) | 4 (7.3) | 1 |  |  |
| Heart failure | 45 (10.9) | 8 (14.5) | 0.495 |  |  |
| CIED | 22 (5.3) | 4 (7.3) | 0.531 |  |  |
| Peripheral arterial disease | 33 (8) | 4 (7.3) | 1 |  |  |
| Cerebro-vascular disease | 32 (7.7) | 6 (10.9) | 0.429 |  |  |
| Respiratory disease | 55 (13.3) | 8 (14.5) | 0.833 |  |  |
| Chronic renal failure | 60 (14.6) | 9 (16.4) | 0.689 |  |  |
| Liver cirrhosis | 42 (10.2) | 5 (9.1) | 1 |  |  |
| Solid neoplasm | 89 (21.6) | 17 (30.9) | 0.126 |  |  |
| Haematological neoplasm | 25 (6.1) | 2 (3.6) | 0.757 |  |  |
| Solid organ transplant | 17 (4.1) | 1 (1.8) | 0.709 |  |  |
| Hematopoietic stem cell transplant | 2 (0.5) | 0 | 1 |  |  |
| Neutropenia (≤500 cells/mL) | 11 (2.7) | 0 | 0.377 |  |  |
| Steroid therapy | 55 (13.3) | 8 (14.5) | 0.833 |  |  |
| HIV infection | 19 (4.6) | 2 (3.6) | 1 |  |  |
| Foci^1^:  -Intravenous catheter  -Osteo-articular infection  -SSTI  -Unknown  -Endocarditis  -Pneumonia  -Urinary tract infection | 166 (40.2)  58 (14)  54 (13.1)  49 (11.9)  27 (6.5)  11 (2.7)  13 (3.1) | 15 (27.3)  3 (5.5)  6 (10.9)  9 (16.4)  11 (20)  4 (7.3)  0 | 0.077  0.088  0.830  0.381  0.002  0.087  0.380 |  |  |
| **High risk foci^2^** | **87 (21)** | **24 (43.6)** | **<0.001** | **2.18 (1.17-4.09)** | **0.014** |
| Septic metastasis | 118 (28.6) | 22 (40) | 0.087 |  |  |
| **Septic shock** | **45 (10.9)** | **20 (36.4)** | **<0.001** | **4.26 (2.17-8.39)** | **<0.001** |
| Need of ICU | 79 (19.2) | 25 (45.5) | <0.001 |  |  |
| Need of MV | 24 (5.8) | 18 (32.7) | <0.001 |  |  |
| AKI at admission | 124 (30.1) | 33 (60) | <0.001 |  |  |
| Baseline creatinine ≥1,10 mg/dL | 187 (45.3) | 39 (70.9) | <0.001 |  |  |
| **AKI (I – III) during admission** | **76 (18.4)** | **25 (45.5)** | **<0.001** | **3.48 (1.86-6.48)** | **<0.001** |
| Persistent bacteraemia | 113 (27.4) | 22 (40) | 0.058 |  |  |
| Active empiric therapy | 337 (83.4) | 48 (88.9) | 0.427 |  |  |
| Empiric therapy:  -aminoglycoside  -glycopeptide  -daptomycin  -PTZ  -carbapenem  -3GC  -cloxacillin | 10 (2,4)  53 (12.8)  135 (32.7)  59 (14.2)  85 (20.6)  94 (22.8)  51 (12.3) | 3 (5.5)  5 (9.1)  14 (25.5)  6 (10.9)  17 (30.9)  11 (20)  6 (10.9) | 0.188  0.519  0.355  0.680  0.085  0.733  1 |  |  |
| Definitive therapy:  -cloxacillin  -cefazolin | 299 (72.4)  114 (27.6) | 43 (78.2)  12 (21.8) | 0.421  0.421 |  |  |

CIED, cardiac implantable electronic device. SSTI, skin and soft tissue infection. ICU, intensive care unit. MV, mechanical ventilation. PTZ, piperacillin-tazobactam. 3GC, third generation cephalosporin.

*30-day mortality could not be assessed in 2 patients; therefore, the analysis included 468 episodes.

^1^ Those with >10 cases are included in the list.

^2^ High risk foci included those with a mortality >15% (endocarditis, pneumonia, and unknown).
